# First-Trimester Diagnosis of Supernumerary Hemivertebra

**DOI:** 10.3390/diagnostics12020373

**Published:** 2022-02-01

**Authors:** Roxana Elena Bohiltea, Ionita Ducu, Bianca Margareta Mihai, Ana-Maria Iordache, Vlad Dima, Emilia Maria Vladareanu, Nicolae Bacalbasa, Alexia-Teodora Bohiltea, Teodor Salmen, Valentin Varlas

**Affiliations:** 1 Department of Obstetrics and Gynecology, “Carol Davila” University of Medicine and Pharmacy Bucharest, 37 Dionisie Lupu, 020021 Bucharest, Romania; nicolaebacalbasa@gmail.com (N.B.); valentin.varlas@umfcd.ro (V.V.); 2 Department of Obstetrics, Gynecology and Neonatology, Filantropia Hospital, 11-13 Ion Mihalache Blv., Sector 1, 011171 Bucharest, Romania; bmmihai@gmail.com; 3 Department of Obstetrics and Gynecology, University Emergency Hospital, 169 Splaiul Independentei Bld., Sector 5, 050098 Bucharest, Romania; 4 Optospintronics Department, National Institute for Research and Development in Optoelectronics-INOE 2000, 409 Atomistilor, 077125 Magurele, Romania; 5 Faculty of Medicine, “Carol Davila” University of Medicine and Pharmacy Bucharest, 37 Dionisie Lupu, 020021 Bucharest, Romania; vladareanu@gmail.com; 6 École Hôtelière de Lausanne, 1000 Lausanne, Switzerland; alexia.bohiltea@ehl.ch; 7 Department of Diabetes, Nutrition and Metabolic Diseases, “Prof. Dr. N.C.Paulescu” National Institute of Diabetes, Nutrition and Metabolic Diseases, 030167 Bucharest, Romania

**Keywords:** hemivertebrae, 11–14 weeks scan, fetal spine, fetal anomalies

## Abstract

Hemivertebra is a common cause of congenital scoliosis and results from a lack of formation of one-half of the vertebral body. This condition is very rare and can present as solitary or as a syndrome component: i.e., the split notochord syndrome, which often implies vertebral defects, from a bifid vertebra to hemivertebrae, or fused vertebrae. We describe a case of supernumerary lateral hemivertebra detected prenatally at 12 weeks of gestation and the ultrasonography specifics that lead to early and accurate diagnosis, monitoring during pregnancy, and follow-up at the 4-year period. The case is presented to specify the importance of an early assessment of fetal spine and diagnosis of various conditions, including hemivertebrae, considering the significant association with other anomalies (cardiovascular, urinary, skeletal, gastrointestinal, and central nervous systems), which are most commonly involved. Moreover, the need to counsel future parents on the risks implied by this anomaly is important for the obstetrician. We underline the inclusion of these types of congenital conditions in high-risk pregnancy because of the frequent association with high cesarean delivery rates, growth restriction, delivery before term, and higher morbidity rates.

## 1. Introduction

As a proportion of 95%, congenital vertebral anomalies are clinically insignificant [1]. However, these conditions can reflect in compression of the spinal cord and are divided into anomalies of the vertebral shape and number. Among all congenital vertebral anomalies, the highest probability of producing neurologic disabilities corresponds to hemivertebrae [2]. Hemivertebra is a common cause of congenital scoliosis and results from a lack of formation of one-half of the vertebral body, having an incidence of 3/10,000 live births [3]. In fact, they are so rare that a recent study [4] found only 324 cases in a time frame of 34 years (1984–2019). This condition can present either as solitary or as a syndrome component: i.e., the split notochord syndrome, which often implies vertebral defects, from a bifid vertebra to hemivertebrae, or fused vertebrae [5] or Alagille syndrome, which is characterized by the liver, heart damage, along with vertebral segmentation defects. Furthermore, hemivertebrae can be a part of the clinical aspect of the Robinow syndrome, dominated by mesomelia or acromesomelia, radioulnar synostosis, brachy-dactylia, syndactyly, ectrodactyly, and craniofacial defects (micrognathia, macrocephaly, depressed nasal bridge, and hypertelorism) [6]. Moreover, hemivertebrae can be found as a congenital skeletal disability within fetal alcohol spectrum disorder [7]. Other syndromes that could imply the presence of hemivertebra are Jarcho-Levin syndrome, OEIS, and VACTERL syndrome. The association with spina bifida is not uncommon [8]. Thus, a chromosomal analysis can be offered even if the incidence of karyotypic abnormalities is low amongst fetuses with isolated vertebral malformation, but there is an increased risk for spina bifida in siblings [9].

Depending on the site of the development defect, hemivertebrae are classified as ventral, lateral, or dorsal. The affected half of the vertebral body may be absent or hypoplastic, respectively, the same for the pedicle or the rib, at the thoracic level. Another classification, depending on the segmentation of the hemivertebra, comprises congenital malformations. These may be adjacent, successive, or unilateral alternant, intermittent leading to a short or long arch of scoliosis, respectively. A hemivertebra can be supernumerary or replace in situ a normal structure; most often, lateral hemivertebra is supernumerary [10].

We describe a case of supernumerary lateral hemivertebra diagnosed prenatally at 12-weeks and the ultrasonography specifics that lead to early and accurate detection, monitoring during pregnancy, and follow-up at the 4-year period.

## 2. Materials and Methods

### Case Report

**Figure 1:**
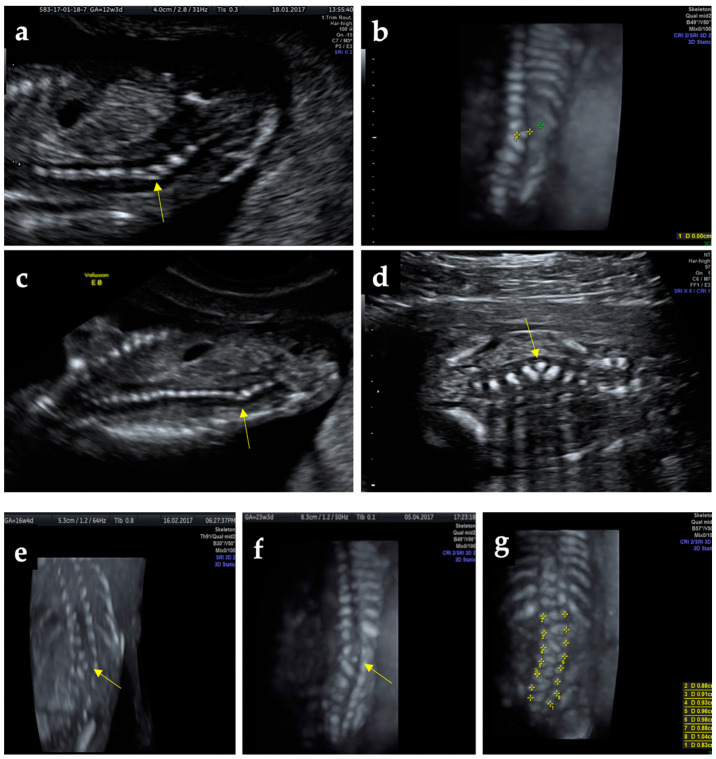

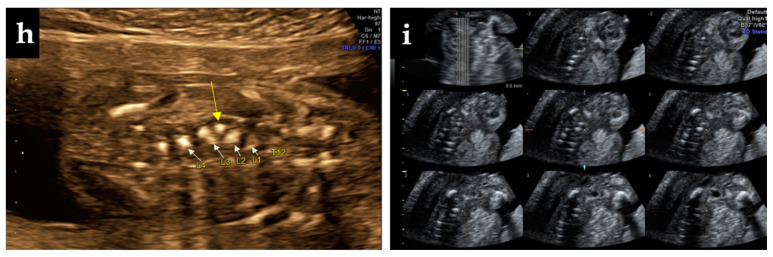
Ultrasound findings: (**a**) suspect angulation of the thoracolumbar spine at 12-weeks show the hemivertebrae (yellow arrow), (**b**) zoom-in above the hemivertebrae in 3D static reconstruction, (**c**) image of the whole spine at 12-weeks; (**d**) clear 2D imaging of the hemivertebrae; (**e**,**f**)—US scans of the fetal spine at 16 and 23 weeks, (**g**) tracing the correspondence of the vertebrae lateral ossification centers, (**h**) numbering and positioning of the vertebrae at 23 weeks on 2D ultrasound sepia mode, (**i**) Tomographic Ultrasound Imaging (TUI)—successive scans of the spine revealing the anomaly.

A 26-year-old pregnant woman with no history of previous births and a spontaneous, uncomplicated abortion at 6 weeks of gestation presents for pregnancy monitoring at 5 weeks of gestation. Family medical history was insignificant. The pregnancy uneventfully evolves through prescribed medication (prenatal vitamins and low dose aspirin 75 mg/day) without folic acid pre-conception prophylaxis. At 12 weeks of gestation during first-trimester screening, combining ultrasound and biochemical test, on ultrasonography (performed with a Voluson E8 machine system from GE Healthcare, Chicago, Illinois, USA, equipped with a transabdominal RAB6-RS convex probe and a RIC5-9-D transvaginal probe), nuchal translucency was in the normal range (1.60 mm), the pulsatility index of ductus venosus was 0.84, nasal bone was present, normal activity and structure of the fetal heart were also normal, and a crown–rump length of 65.6 mm corresponding to 12 weeks and 6 days of gestation was measured. In the coronal plane, at the L3–L4 spine level, there was observed a triangular hyperechoic structure on the lateral side of the spinous processes, located between vertebrae normally positioned and determining a sharp angulation of the column spine with the concavity to the left (Figure 1a–d); all the other scanning planes (sagittal and transverse) appeared normal. Repeated ultrasound examinations at 16, 17, and 23-weeks using Tomographic Ultrasound Imaging (TUI) and three-dimensional (3D) reconstruction during pregnancy revealed no progress of angulation, with no lordosis or scoliosis and without any adjacent aspect suggestive of a neural tube defect (Figure 1e–i and Supplementary Video S1). A careful morphologic inspection did not find other associated malformations. The suspected diagnosis of supernumerary hemivertebra was confirmed at that stage of the pregnancy by two other ultrasonography specialists. The combined test at 12 weeks and 6 days of gestation placed the patient in the low-risk group for chromosomal anomalies. Because the patient could not afford an additional test, the fetal karyotype was not performed. After counseling regarding the possible complications and associated anomalies, the future parents opted for the continuation of pregnancy.

At 38 weeks of gestation, in the context of the spontaneous onset of labor and spontaneous ruptures of the fetal membranes, an apparently healthy male fetus weighing 2350 g, 46 cm, Apgar score 9 was extracted through cesarean section.

The infant remained under the supervision of the pediatric orthopedic surgeon and neurosurgeon, having a normal neuro-physical development up to the age of 4 years and 6 months.

**Figure 2:**
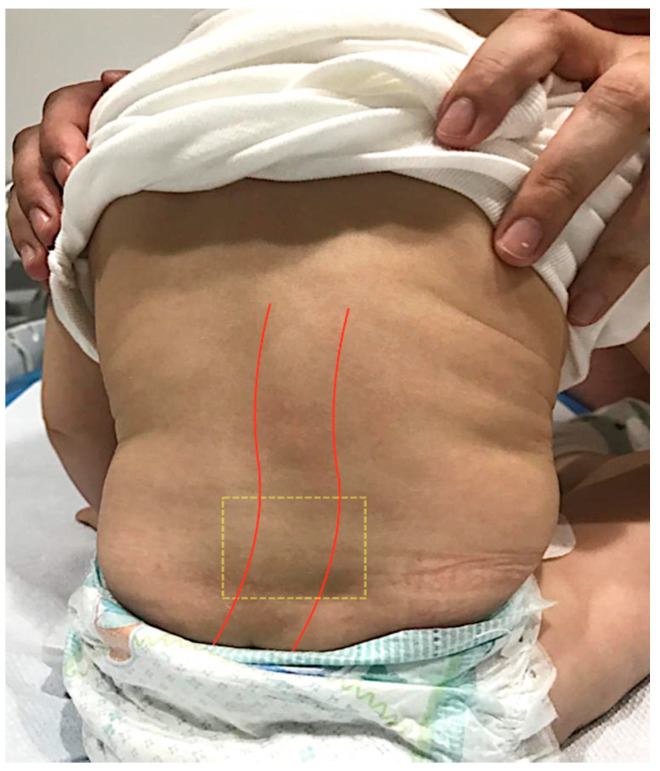
At 8 months after delivery, a physical examination identified a mild scoliosis (red line) and rectangular zone (interrupted yellow line) in front of the transverse process L3–L4.

At 8 months after delivery, a routine consultation revealed the presence of an approximately 3 × 4 cm wide plaque on the right of the spine, without reflection at the skin, with mild scoliosis, suggesting the welding of the transverse process L3–L4 (Figure 2).

**Figure 3:**
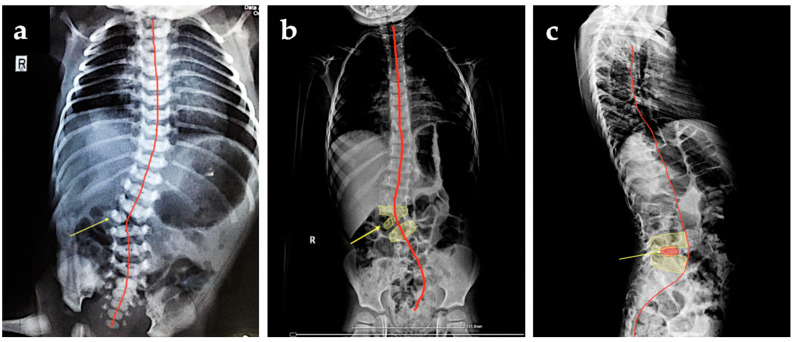
X-ray findings: (**a**) anteroposterior face of the child’s spine at 8 months after delivery indicating hemivertebra (yellow arrow); (**b**) anteroposterior X-ray face of the patient spine at 4-years-old depicting hemivertebra (yellow arrow) and scoliosis (red line), (**c**) lateral profile of the spine focused on the thoracolumbar section revealed the hemivertebra (yellow arrow), and curvature of the spine (red line).

X-ray postnatal evaluation confirmed the early prenatal ultrasonographic diagnosis (Figure 3a). The parents refused further complementary imaging (MRI) despite the medical advice. The X-ray performed in the current year revealed in the anterior-posterior face X-ray of the thoracolumbar child’s spine, the hemivertebra, along with a mild scoliosis (Figure 3b,c).

## 3. Discussion

The first supernumerary skeletal anomaly we could find, historically wise, was a report of a supernumerary first rib published in the Lancet on 29 December 1860 [11]. Since then, other reports describing supernumerary ribs have been published [12,13,14], whereas reports on supernumerary vertebra are very rare [15]. With a low incidence, hemivertebrae is usually found at the thoracic level [16], lumbar portion, and thoracolumbar region [17], having a slightly increased prevalence in female fetuses with a ratio of male to female of 0.68 [18]. In our case, this condition was present in a male fetus, and it was placed in the lumbar region. Placement of the hemivertebrae is important: when localized at the thoracic level, scoliosis or kyphosis is recommended to be surgically corrected, but when it occurs in the low lumbar region, surgical intervention is not necessary (deformity of the spine is minimal and usually not observed) [19]. By searching the literature regarding the incidence, diagnosis, and treatment of supernumerary hemivertebra, we noticed that the number of reported cases is low, and the number of patients diagnosed prenatally in the first trimester is even lower [20]. The gestational age at which it is usually diagnosed is 20–28 weeks [17,18,20]. However, some studies have been able to prenatally diagnose hemivertebra between 15 and 23 weeks for a number of 3 patients out of 26 [3] and between 11–14 weeks in a number of 10 patients, respectively [21]. This last study [21] indicates that only two fetuses were safely delivered, while the other cases were either terminated (seven cases) or miscarried (one case). Thus, due to the rare incidence of this abnormality and to the fact that most parents decide to terminate the pregnancy, it is difficult to assert the natural course of the condition: could the fetus develop normally and be safely delivered, or will it end in a miscarriage? Furthermore, the right moment for the surgical intervention for the correction of the spinal abnormality is unknown. This makes it difficult to counsel the parents, especially when the chromosomal abnormalities do not correlate with the hemivertebrae (non-isolated hemivertebrae could indicate abnormal karyotype) [21].

Prenatal diagnosis of musculoskeletal anomalies is challenging to perform because the vertebral column undergoes structural and morphological changes between 9 and 22 weeks, with separation of the vertebral arches up to 19 weeks [22]. Transvaginal ultrasound has a better resolution than transabdominal sonography, but in the first trimester, the coronal view cannot always differentiate between normal vertebrae and a hemivertebra abnormal presence unless the scanning is very detailed. However, using 3D ultrasound, the specialist can vary the contrast between bones and tissue (by using contrast enhancement rendering algorithms) to perform a thorough spine evaluation [23]. In the ultrasound diagnosis, the images show: (1) the deformity of the spine accompanied by blurring of the vertebral arch lesion with obvious loss of the ossification center; (2) the cross-section of the vertebral body has an irregular shape, with blurred edges; (3) the 3D ultrasonic image of the vertebral column relays the exact localization of the anomaly in the shape of a triangle, causing the contralateral deviation of the spine [17,24,25]. In our case, we detected an extra ossification center, not an ossification center loss, which was guided further through an ultrasonographic scan of the spine.

Spinal development that resumes in primitive vertebrae begins around six weeks of gestation, continuing with the fusion of the chondrification centers in the seven–eight weeks of pregnancy, forming a primary ossification center. Supernumerary lateral hemivertebra results from a supernumerary, unpaired, undeveloped chondral center of unknown cause. As a result, supernumerary hemivertebra can be diagnosed in the early stages of the pregnancy on a detailed scan of the spine. Differential diagnosis includes other vertebral anomalies, such as bloc vertebra, butterfly vertebra, and wedge-shaped vertebra, frequently associating an unfavorable outcome.

The association with other musculoskeletal malformations of the spine, limbs, and ribs is very common [3], and from the extra-musculoskeletal anomalies, cardiac malformations and anomalies of the genitourinary tract are the most frequently encountered.

The management of such cases is influenced by the clinical impact of the hemivertebra on the spinal cord, the degree of scoliosis if present, the complexity of the associated malformations if present and diagnosed, and of course, the wish of the future parents. Usually, when scoliosis is present, the typical management is resection [26,27,28,29,30].

Usually, isolated hemivertebra associates a good prognosis, and in 25% of cases, there is no progression to scoliosis or of scoliosis if present [31]. However, an early diagnosis offers the possibility of a meticulous following of the pregnancy, with serial ultrasonographic evaluations, monitoring of the fetal growth, being known that fetal growth restriction has an increased incidence amongst these cases. Our case is proof that supernumerary hemivertebra is associated with intrauterine growth restriction.

A particular delivery for the cases with isolated hemivertebra is not required, even though, according to reported data—Table 1, it presents a higher rate of cesarean birth [8], prematurity, and neonatal death. A study of 27 hemivertebra cases [9] concluded that a prenatal diagnosis of isolated hemivertebra improves the outcome, these cases presenting a good prognosis, but if associated with other anomalies, the survival rate reduces to 50% and zero if accompanied by oligohydramnios. Prenatal diagnosis should rule out life-limiting spinal abnormalities that may be associated with antenatal and intrapartum complications [32].

Our case is illustrative for the importance of an early and correct diagnosis of hemivertebrae. Due to the great proportion of other associating anomalies involving cardiovascular, urinary, skeletal, gastrointestinal, and central nervous systems (among the common ones), the materno-fetal specialists and gynecologists have to put a lot of care into communicating the results to the parents and informing them of the risks and complications associated with this anomaly (such as high rates of cesarean delivery, growth restriction, delivery before term, and higher morbidity rates).

## 4. Conclusions

We conclude that a careful scan of the entire spine should be highly recommended in the first-trimester ultrasound screening, considering the reported association with other anomalies. Given its higher resolution and more detailed scan, we would like to emphasize that the ultrasound method of choice of screening for spinal abnormalities in the first trimester should be the transvaginal approach.

## Figures and Tables

**Table 1:** Summary of the characteristics of other cases of supernumerary vertebra, compared to our case.

Reference	GA at Diagnostic	Postnatal Status	Ultrasound Findings
9 cases [17]	16–27 weeks	TOP	Localization of hemivertebra, the condition of the lesion, abnormalities in the internal organs (e.g., absence of kidneys or ribs), blurring of the ossification center, presence of scoliosis and open spine bifida
1 case [19]	postnatal	well	Small asymmetrical appearance of a vertebral body and a focal defect on either side of the vertebral column—the fetus was diagnosed postnatal
10 cases [21]	11–14 weeks	80% TOP; 20% well	Location of hemivertebrae and co-existing anomalies
2 cases [22]	18–21 weeks	TOP	Abnormal curvature of the spine, multiple hemivertebrae, thoracic kyphosis
1 case [23]	20 weeks	TOP	Abnormal deviation of the spine, with multiple fused hemivertebrae and absence of ribs
Our case	12 weeks	well	Triangular hyperechoic structure at lumbar level with no other abnormalities

GA–gestational age; TOP–termination of pregnancy.

## Supplementary Materials

The following supporting information can be downloaded at: https://www.mdpi.com/article/10.3390/diagnostics12020373/s1: Video S1: Hemivertebrea1.
